# Coculture with Neural Stem Cells May Shift the Transcription Profile of Glioblastoma Multiforme towards Cancer-Specific Stemness

**DOI:** 10.3390/ijms24043242

**Published:** 2023-02-07

**Authors:** Manjusha Vaidya, Sandeep Sreerama, Maxine Gonzalez-Vega, Jonhoi Smith, Melvin Field, Kiminobu Sugaya

**Affiliations:** 1 Burnett School of Biomedical Sciences, College of Medicine, University of Central Florida, Orlando, FL 32816, USA; 2 Orlando Neurosurgery, AdventHealth Neuroscience Institute, Orlando, FL 32803, USA

**Keywords:** extracellular vesicles, cancer stem cells, glioblastoma multiforme, neural stem cells, ABC transporter gene, stemness genes

## Abstract

Glioblastoma multiforme (GBM) possesses a small but significant population of cancer stem cells (CSCs) thought to play a role in its invasiveness, recurrence, and metastasis. The CSCs display transcriptional profiles for multipotency, self-renewal, tumorigenesis, and therapy resistance. There are two possible theories regarding the origin of CSCs in the context of neural stem cells (NSCs); i.e., NSCs modify cancer cells by conferring them with cancer-specific stemness, or NSCs themselves are transformed into CSCs due to the tumor environment created by cancer cells. To test the theories and to investigate the transcriptional regulation of the genes involved in CSC formation, we cocultured NSC and GBM cell lines together. Where genes related to cancer stemness, drug efflux, and DNA modification were upregulated in GBM, they were downregulated in NSCs upon coculture. These results indicate that cancer cells shift the transcriptional profile towards stemness and drug resistance in the presence of NSCs. Concurrently, GBM triggers NSCs differentiation. Because the cell lines were separated by a membrane (0.4 µm pore size) to prevent direct contact between GBM and NSCs, cell-secreted signaling molecules and extracellular vesicles (EVs) are likely involved in reciprocal communication between NSCs and GBM, causing transcription modification. Understanding the mechanism of CSC creation will aid in the identification of precise molecular targets within the CSCs to exterminate them, which, in turn, will increase the efficacy of chemo-radiation treatment.

## 1. Introduction

Glioblastoma Multiforme (GBM) is a cancerous primary brain tumor with a devastating prognosis due to the lack of a curable treatment in spite of extensive therapeutic research. GBM tumors are highly diverse, with a mixed population of cancer cells, cancer stem cells (CSCs), and normal neural stem cells (NSCs). NSCs tend to infiltrate the tumor and migrate through the central nervous system parenchyma along with cancer cells [1]. GBM-specific CSCs are a relatively modest population of specialized tumor cells with self-renewal and unlimited propagation abilities. They are chiefly responsible for conventional therapy resistance, making GBM impossible to treat. Genetic heterogeneity of tumor cell populations and the emergence of intrinsically therapy-recalcitrant CSCs are thought to be the most critical reasons for GBM recurrence and spread [2]. CSCs also contribute to GBM tumor initiation, maintenance, and propagation [3]. Just like NSCs, CSCs display expression profiles of embryonic stemness genes, the main drivers of pluripotency [1,4]. The cancer-specific stemness of the CSCs is closely associated with ATP binding cassette (ABC) transporter pathways and elevated efficiency of DNA repair [5,6,7]. The membrane transporters belonging to the ABC transporter gene family are upregulated in CSCs and promote drug efflux in therapy-targeted cells [7,8]. DNA repair protein O6-methylguanine-DNA-methyltransferase (*MGMT*), with its increased expression, is implicated in the chemotherapy drug Temozolomide (TMZ) in GBM [5]. Therefore, we have investigated the genes involved in embryonic stemness, drug efflux, and DNA repair to study the transformation of neural stem cells or GBM tumor cells into CSCs.

Based on the shared identity among various gene expression profiles, GBM CSCs are considered to be the cancerous variation of NSCs, where both the cell types follow common signaling pathways of neural development. Although they share stem-cell-specific biomarkers and gene expressions, the origin of CSCs has not been successfully pinned down to oncogenic mutations in NSCs or gain of stemness in cancer cells [3]. Xu et al. have shown that a non-neural cell becomes tumorigenic due to the property of neural stemness [9]. According to their research, the removal of transcriptional repressor results in the loss of cell identity and a gain of NSC property as well as tumorigenicity in intestinal stem cells [9]. To understand the role of NSCs in CSC creation, the present study evaluated the vulnerability of NSCs to malignant transformation, along with their ability to modify the cancer cells into CSCs.

In the context of CSCs as the means of cancer initiation and sustained propagation, a hierarchical model has been proposed. In this model, highly tumorigenic stem cells with cancer-specific stemness occupy the topmost position for creating differentiated cancer cells as well as maintaining the copy of the CSC [10]. For the creation of CSCs, we have focused on the participation of NSCs in the transformation process. There are two possible scenarios where NSCs can be involved in CSC inception: (i) GBM cancer cells gain stemness under the influence of NSC-secreted signals, and; (ii) upon exposure to the cancerous tumor environment, the NSCs themselves transform to CSCs due to their susceptibility to malignant transformation. The cancerous surroundings or tumor microenvironment can trigger cancer-driving mutations in NSCs to transform them into CSCs [11]. A direct implication of NSCs in CSC creation has not yet been researched.

In both the theories, molecular signaling between NSCs and cancer cells is necessary for transformation into CSCs. In gliomas, such intercellular communication occurs via multimodal pathways involving direct cell–cell contact, connecting nano- or micro-tubes and extracellular vesicles (EVs) [12]. The bilateral talk involves immune signaling protein molecules, cytokines, chemokines, angiogenic growth factors, hormonal/non-hormonal growth factors, and other extracellular molecules comprising nucleic acids and metabolites [13,14,15,16,17]. Diffusible factors secreted by GBM adversely affect NSCs via induction of oxidative stress [18]. GBM also uses non-canonical pathways for unconventional protein secretion to maintain the tumor [19]. On the other hand, NSCs secrete immune-modulatory factors with a regenerative effect, along with chemokines, cytokines, and proteins involved in cell signaling pathways [20,21]. Although the crosstalk between cancer and noncancer cells transpires via functionally active, membrane bound, or membrane-free signaling molecules, specifically in GBM, EVs are a vital courier for the communication between tumor cells and their environment. In GBM, EVs have a significant role in cancer progression and therapy resistance [13,22]. EVs, the lipid membrane-bound nano-vesicles secreted by normal as well as cancer cells, are a heterogenous population comprising exosomes (30–150 nm diameter), micro-vesicles (100–1000 nm diameter), and apoptotic bodies (800–5000 nm diameter). EVs can modify innate cellular programs and participate in the communication between a parent cell and its surroundings. The tumor-derived EVs carry ontogenically modified nucleic acids and proteins capable of initiating cancerous or pre-cancerous pathways in the recipient cell [23,24]. GBM-derived EVs are known to transport the macromolecular cargo comprising cancer-specific single and double-stranded DNA, microRNA, long noncoding RNA, mRNA, signaling proteins (including enzymes and ligands, as well as receptors), and lipid rafts for intercellular communication [25]. The functionally active cargo of EVs has a pleiotropic effect on the receiving cell [26]. Although GBM is known to employ various means of communication to promote growth and invasion, EVs are uniquely suited to distribute cancer-specific vesicular cargo more effectively in the tumor microenvironment, and also to distant sites [27]. EVs carry a distinctive molecular signature of their parent cells and they can cross the blood–brain barrier (BBB) to exert a paracrine effect on distant cells in addition to their local tumor microenvironment. Glioblastoma cells secrete EVs carrying pro-permeability factors that can induce permeability through the BBB [28]. Tumor cells can thus modify their own microenvironment via EVs [29,30]. On the other hand, NSC-secreted EVs are enriched with stem cell-specific cargo comprising nucleic acids and proteins that specifically participate in neuroprotection and neural cell differentiation [31,32]. EVs from normal, non-cancerous cells efficiently communicate with tumor cells to regulate tumor progression [22,30]. EVs are also implicated in tumor drug resistance via direct transportation of chemotherapeutic drugs out of the cells, thereby reducing their concentrations at the target making the treatment less effective [22].

Because the diffusible factors, extracellular macromolecules, and the EV-mediated bilateral communication brings about paracrine signaling in the tumor environment, we have cocultured GBM cells and NSCs in the same well of a multi-well plate. The co-culture setup is such that the two cell lines have no direct contact with each other, although they share the culture media. Separated by a cell insert with a membrane of 0.4 µm pore size, the cells have a mutual exposure to the EVs and diffusible macromolecules secreted by the other cell line. Because some of the secreted molecules are transported via EVs, in a separate experiment, we also confirmed their internalization by the recipient cells. The reciprocal effect of factors secreted by NSC and GBM, and their conveyance via EVs will underscore the molecular mechanism of CSC formation, identifying the unique molecular targets to improve GBM treatment.

## 2. Results

### 2.1. EV Internalization

**Figure 1:**
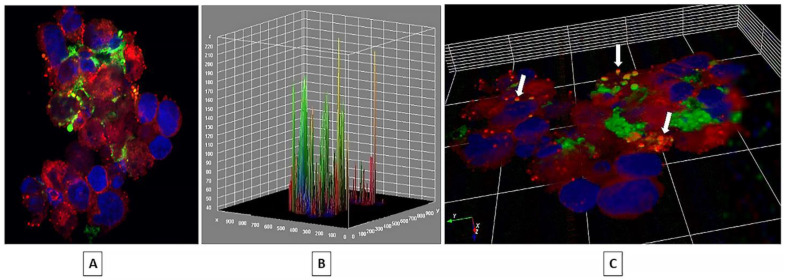
Composite Confocal image of a GBM cell cluster exposed to NSC-derived EVs. GBM cells are dyed with DiI, a lipophilic dye (Red). NSC EVs are green due to their parent cells being dyed with DiO, another lipophilic dye (Green). Nuclei are stained with DAPI (Blue) [33]. (**A**) A 2D image of the NSC EVs internalized in GBM cells. (**B**) A 3D Surface Plot of the same image created using ImageJ software. The yellow peaks represent the colocalization of the red signal from GBM cells and the green signal from the NSC EVs. (**C**) A 3D rendering of the image created using Icy, an open-source bioimage informatics platform [34]. The white arrows point to the colocalization signal of red and green colors indicating internalized EVs.

Confocal microscopy images and the Z-stacks confirm uptake of mutual EVs by both GBM and NSCs (Figure 1). DiI-dyed red cells of GBM are seen with green specs of NSC-secreted EVs inside the cells, and DiO-dyed green NSCs have red, GBM-secreted EVs internalized by the cells.

### 2.2. Relative Expression of Genes in Coculture

In the relative expression analysis of gene transcripts, the cocultured GBM showed a significant upregulation of the ABC transporter gene, ABCG2. Concurrently, the DNA repair gene, MGMT, was also highly upregulated. Where ABCB1 expression had no significant change in GBM upon coculture, the expression of ABCC1 was notably lower. Neural progenitor and stemness markers CD133, CD44, along with CD9 and TUBB3 had a notable increase in expression in cocultured GBM. Where CD44, a CSC marker, showed slightly higher expression in cocultured GBM, another CSC marker, SOX9, was highly upregulated. NANOGP8, a retro-onco copy of embryonic stem cell gene NANOG, another embryonic stem cell marker-SOX2, and GFAP showed no significant changes in expression in GBM upon coculture. With the exception of upregulated expression of CD9, the cocultured NSCs showed either downregulation or no significant change upon coculture.

**Figure 2:**
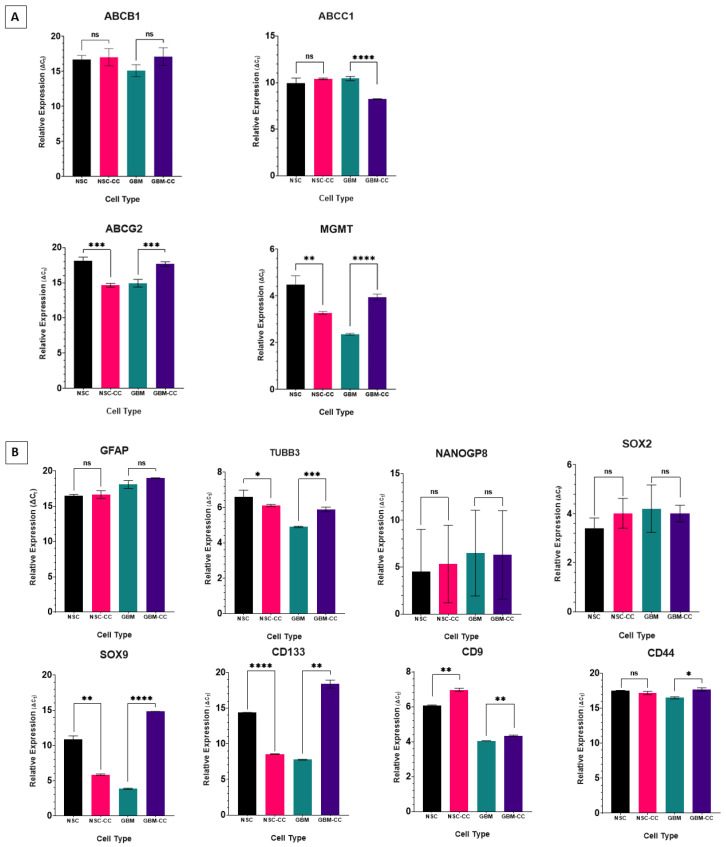
Relative expression graphs for GBM and NSC cocultured cells. (**A**) *ABC transporter cluster:* ABCB1, ABCC1, ABCG2, and MGMT (DNA repair). (**B**) *Stemness/CSC cluster:* CD9, CD133, CD44, GFAP, TUBB3, NANOGP8, SOX2, and SOX9. NS: no statistically significant, *: *p* < 0.05, **: *p* < 0.01, ***: *p* < 0.005, ****: *p* < 0.001.

The relative expression analysis of genes categorized as a stemness cluster and drug efflux or ABC transporter cluster revealed that the GBM, with an upregulation for almost all the genes in the clusters, shift towards stemness, whereas NSCs, with the downregulation of the same genes, show characteristics of a differentiated cell line. Table 1 and Figure 2 sum up the relative expressions of all the gene transcripts in both the cocultured cell lines.

### 2.3. Gene Expression in GBM CSCs

**Figure 3:**
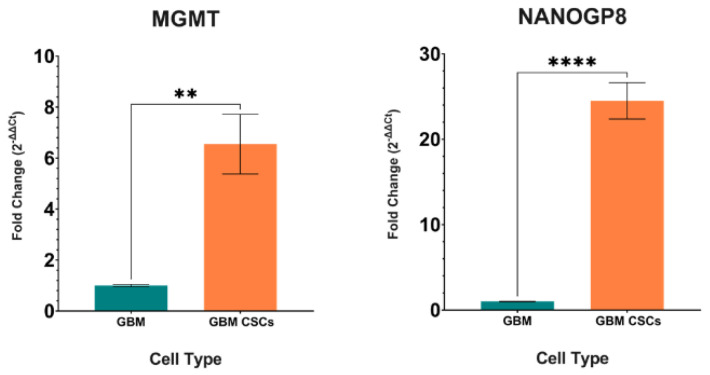
Fold change expression graphs for GBM and GBM CSCs. DNA repair gene MGMT and the CSC-specific gene NANOGP8 show significantly higher expressions in CSCs as compared to the GBM, which contains a mixed population of cancer cells along with CSCs. **: *p* < 0.01, ****: *p* < 0.0001.

To have a baseline value for gene transcription within the CSCs, MGMT and NANOGP8 transcripts were quantitatively analyzed in comparison with GBM. The two genes with high expression are representatives of the stemness traits in CSCs. The higher expression of the two gene transcripts indicates that in GBM neurospheres with a mixed population of cancer cells and CSCs, the latter ones have a distinguishing transcription profile ascribed to stemness (Figure 3).

## 3. Discussion

In addition to embryonic stem cell (ESC) markers such as NANOG and SOX2, GBM-specific CSCs express neural progenitor markers for stemness that include CD133, CD44, and GFAP [10]. When transplanted in a non-indigenous environment of an immunodeficient mouse model, only neural stem cells and neural progenitor cells display tumorigenicity, but not the non-neural stem cells [9]. Therefore, CSCs of GBM possessing the same neural progenitor transcription signature is considered to be of NSC origin. A cancer stemness-specific oncogene, NANOGP8, is expressed in CSCs. According to Zbinden et al., specifically in GBM, the ES gene NANOG is expressed in the form of its retrogene NANOGP8 [35]. CSCs share a gene expression profile comprising CD133 and TUBB3 with NSCs. This is another reason that the stem cells of GBM are thought to originate from the NSCs [35]. Given this background, we have studied the expression of ES genes and neural progenitor markers via quantitative analysis of their transcripts. We found that NSCs secreted signals affect the GBM transcriptome and upregulate the expression of TUBB3 and CD133, although ES gene expression shows no significant alterations. The finding affirms that NSCs are capable of inducing transcription, potentially leading to CSC tendencies in cancer cells.

Though CSCs and NSCs share functionally similar traits of differentiation and sustained proliferation, they differ in many aspects, especially in terms of maintaining a stable cell population. NSCs of a normal brain possess stem-cell-specific properties of self-renewal and multipotency with a stringent equilibrium among cell proliferation, differentiation, and apoptosis [36]. The stemness of the normal NSCs is maintained by the interaction between the innate cellular programs and the external factors, maintaining normal homeostasis of the cell number [37]. CSCs, on the other hand, adapt abnormal genetic profiles through transcriptional dysregulation. Most importantly, the effect of the tumor microenvironment and the loss of homeostatic properties make tumorigenic CSCs deviate from their normal counterpart [38].

Transmembrane glycoprotein CD44 and members of the SOX transcription factor family, SOX2 and SOX9, are implicated in the CSC function of GBM cells. They are considered to be CSC markers [39,40,41,42]. CD133-negative GBM cells are shown to possess upregulated CD44 displaying CSC traits [39]. CD44 is a transducer of cancer-specific signals from the cellular environment and a known cancer stemness and metastasis regulator. Similarly, CD9 is attributed to CSC properties, including tumor formation and the maintenance of tumor cell population [7]. In this study, NSC-secreted EVs may possibly provide ready-made factors to GBM cells to block pro-differentiation signals that result in an upregulation of expression of stemness genes CD9 and CD44 along with TUBB3, CD133, and SOX9, leading the cancer cells to shift the transcription to cancer-specific stemness. Conversely, the downregulation of neural stemness markers such as SOX9 indicates the differentiation effect that the GBM-secreted EVs may have exerted on the NSCs.

GBM-specific CSCs are categorized into pro-neural and mesenchymal subtypes with different molecular signatures, e.g., where CD133 and SOX2 are the markers for the pro-neural type of CSCs, CD44 belongs to the mesenchymal subtype [43]. In this study, the upregulated relative expression of CD133, with a significant *p* VALUE <0.005 as compared to the *p* VALUE <0.05 for CD44, underscores the effect of NSCs, the stem cells of neuronal lineage.

Normally, TUBB3, a neuronal marker, is not expressed in mature glia. However, under neoplastic conditions, its expression ceases to be neuron-specific [44]. Importantly, in central nervous system (CNS) cancers, TUBB3 expression is vital and associated with a higher histological grade of malignancy [45]. An elevated TUBB3 expression in cocultured GBM signifies its shift in the direction of cancer stemness.

O6-methylguanine-DNA methyl-transferase (MGMT) is a DNA repair protein whose expression and promoter methylation status are crucial for chemotherapy involving alkylating agents. MGMT counteracts the effect of TMZ, and its silencing improves the efficacy of TMZ treatment in GBM [46,47]. GBM stem cells show high expression of this gene, which is a reason for TMZ resistance [48]. In our experiments, CSCs show higher MGMT expression along with significant elevation of cancer stemness marker NANOGP8 as compared to GBM. A significantly elevated MGMT expression in GBM upon coculture with NSCs also points to the GBM’s shifting towards stem cell characteristics. In cocultures, GBMs do not show a change in NANOGP8 expression. No change is detected in SOX2 expression either. It is possible that the embryonic stemness gene expression gears up later on in the CSC formation process, where other CSC marker genes such as SOX9, CD44, stemness marker CD133, and the ABC transporter gene ABCG2, with their upregulated expressions, kickstart the process. It is also possible that the embryonic stemness genes do not have to be upregulated, and their base-level expressions are enough to initiate the transformation process for cancer-specific stemness. SOX9, a player in CSC sustenance, is also responsible for cell senescence in gliomas and works synergistically with SOX2. The silencing of SOX9 downregulates the expression of CD133 and SOX2 [41]. In our research, although SOX9 and CD133 are highly upregulated, SOX2 shows no significant alteration upon coculture of GBM with NSCs. However, an upregulation of cancer stemness markers such as SOX9, CD44, and CD9 implies that GBM is potentially moving towards cancer-specific stemness in the presence of NSCs.

In addition to MGMT expression, TMZ resistance of CSCs in GBM is attributed to the overexpression of efflux proteins that belong to the ABC family of membrane transporters which confer stemness properties to cancer cells [49]. ABC transporters are membrane pumps that use ATP hydrolysis to transport substrates such as TMZ across a cellular membrane and also hinder drug uptake, thereby imparting chemotherapy resistance to CSCs [7,50]. ABC transporters are not only the participants in chemotherapy drug efflux in CSCs, but are also postulated to be responsible for exporting cell signaling molecules that aid tumorigenesis [8]. For this reason, in the transcript expression analysis, we included the three members of the ABC transporters, ABCB1, ABCC1, and ABCG2, identified in the CSCs of tumors [49]. Each transporter is able to transport a specific set of drugs [7]. We found that the transcription of ABCB1 was unaltered, but ABCC1 was severely downregulated. We also detected significantly elevated expression of ABCG2 in GBM cocultured with NSCs. This result is noteworthy because high expression of CD133 is correlated to higher expressions of ABC transporters, specifically ABCG2, and as a consequence, increased drug resistance. Correspondingly, upregulation of ABCG2 is known to increase the population of CD133-positive cells [7].

Overall expression analysis showed an upregulation of not all but most of the genes involved in cancer stemness in GBM cells upon cocultures with NSCs. The increased expression of CD133, CD9, and CD44, along with SOX9, TUBB3, MGMT, and ABCG2, may have led the GBMs on a path of acquiring cancer-specific stemness. NSCs secrete factors such as Interleukin 6 (IL-6) and VEGF [21]. Where IL-6 signaling is known to promote CSC phenotype in gliomas, vascular endothelial growth factor (VEGF) enhances tumorigenesis and cell proliferation [51,52]. The NSCs, on the other hand, have shown the downregulation of the stemness genes indicating a potential differentiation due to the GBM-derived factors. For example, the GBM is known to secrete pro-inflammatory cytokines such as IL-1 that promote neuronal differentiation [53,54,55]. The important note in this study is that GBM and NSC are cocultured but separated by a cell insert that has a membrane with a pore size of 0.4 µm. Therefore, only cell-secreted substances smaller than 0.4 µm, including EVs that pass through the membrane, influenced the other cells. Although the confocal images showed a successful uptake and internalization of EVs, the factors influencing transcription may not be limited to the cargo transported by EVs.

GBM cells are known to gain stem-like properties under pathological conditions. For example, the stress of an acidic tumor microenvironment and associated hypoxia promote a CSC phenotype [3,56,57]. Therapeutic agents, including TMZ, are also known to induce a CSC phenotype in cancer cells [3]. Hypoxia, inflammation, and necrosis influence CSC progression [58]. It is possible that genes like ABCC1, GFAP, NANOGP8, and SOX2, that show no significant (NS) alternated expression in GBM upon coculture may show variation over time or with the provision of suitable physiological conditions.

## 4. Materials and Methods

CSCs derived from a human primary GBM tumor cell line were cocultured with commercially available NSCs. Separated by a cell insert with a membrane of 0.4 µm pore size, the two cell lines were seeded in the same well but lacked direct contact with each other. The culture media flowed freely between the cell lines through the membrane allowing only the EVs and the cell-free signaling molecules secreted by each cell line to pass through. We evaluated the exchange and the subsequent internalization of the EVs in a separate experiment.

### 4.1. Cell Culture

Human neural stem cells (NSC) were procured from Lonza (catalog # PT2599). Previously untreated fresh GBM tumor cells with a mixed population of differentiated cancer as well as CSCs were obtained from an otherwise healthy adult patient via craniotomy with preoperative imaging suggestive of GBM and intraoperative frozen section confirming the diagnosis. Institutional Review Board-approved informed consent for the research study prior to the surgery was obtained and Health Insurance Portability and Accountability Act (HIPAA) regulations were strictly followed. The cell lines have been characterized in detail previously [59]. Tumor cells were dissociated and grown in in vitro culture. Both NSC and GBM cell lines were grown in suspension cultures, where they formed neurospheres. CD133 antibody conjugated magnetic microbeads were used to separate the cancer stem cells (CSCs) from the GBM neurospheres following the manufacturer’s protocol (Miltenyi Biotec, Bergisch Gladbach, Germany, CD133 MicroBead Kit—Hematopoietic, catalog #130-100-830). With the help of LS columns (Miltenyi Biotec, catalog #130-042-401), the positively selected CD133^+^ cells were collected and grown in the suspension culture. The growth media contained Heparin 5000 U (0.5 U/mL), EGF 20 ng/mL, bFGF 20 ng/mL, and 2% B27 stock mixed in DMEM/F12. By using culture media that does not require fetal bovine serum, there was no confounding bovine EVs in the harvested cell lines created.

### 4.2. Coculture

**Figure 4:**
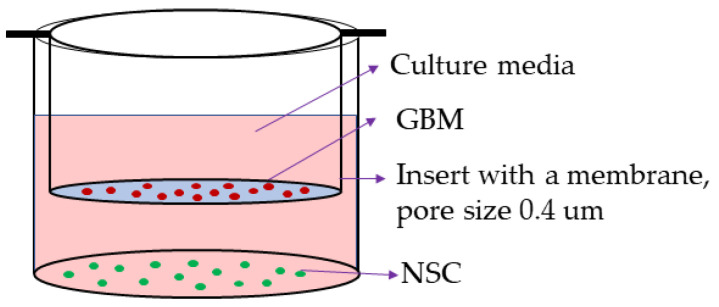
Coculture setup. A single well representation from a 6-well plate. GBM cells were cultured in the insert while the NSCs were positioned at the bottom of the well outside of the insert. Cells were unable to pass through the 0.4 µm pore size of the cell insert membrane, only letting EVs and signaling molecules pass through.

Both the cell lines were grown in NSC media. An initial volume of 4 mL of NSC media was added to each well of a 6-well non-tissue culture-treated plate. A cell culture insert (Falcon™ Waltham, MA, USA, Cell Culture Inserts Catalog #08-771) having a membrane pore size of 0.4 µm was immersed in the media, and the plate was incubated overnight at 37 °C to condition the membrane. Neurospheres of NSC and GBM cells were singularized through repeated and gentle aspiration, followed by treatment with cell dissociation reagent Accutase (Gibco™ Waltham, MA, USA, StemPro™ Accutase™ Cell Dissociation Reagent, catalog #A1110501). 0.1 × 10^6^ cells of GBM were seeded in the cell culture insert, whereas approximately the same number of NSCs were seeded at the bottom of the plate. Cells were cocultured at 37 °C with 5% CO_2_ for eight days with 2.5 mL media added on day 4. Throughout the coculture period, no conditioned medial was removed from the well. Therefore, over a period of 8 days, the cells were cultured in a total of 6.5 mL of nutrient media. Cell setup for each condition was run in triplicate. Figure 4 depicts the cell coculture setup.

### 4.3. RNA Extraction

With careful aspiration, cell culture media and GBM cells from the cell culture insert were transferred to a 15 mL conical tube. The membrane of the cell insert was gently scrubbed with the end of a serological pipette and rinsed with culture media to remove any attached GBM cells. Similarly, NSC cells from the well, along with the media, were moved to a separate tube. Both cell lines were centrifuged at 0.3 rcf for 5 min at room temperature. The supernatant was then discarded, and the cells were resuspended in 500 µL of ice-cold 1x PBS, followed by another centrifugation at 0.3 rcf for 5 min at 4 °C. The supernatant was removed, and the cell pellet was resuspended in 500 μL of TRIZOL reagent. GBM and NSC cells were processed separately for RNA extraction following the same protocol. Total RNA was extracted using the Direct-zol RNA extraction kit (Zymo Research, Irvine, CA, USA, catalog #R2050), following the manufacturer’s protocol, including the in-column DNase treatment to remove genomic DNA contamination. All the centrifugations were performed at 12,000 rcf for 30 s. The extracted RNA was stored at −20 °C for further use.

### 4.4. cDNA Synthesis

A total of 2.5 µg of RNA was converted to cDNA with oligo-dt primer using the SuperScript™ III First-Strand Synthesis System (Invitrogen, Waltham, MA, USA, Fisher scientific catalog #18080051). RNA was incubated with Oligo(dt) at 65 °C for 5 min, followed by an RNA conversion reaction that proceeded as follows: 25 °C for 10 min, 50 °C for 50 min, and 85 °C for 5 min and chilled on ice (4 °C). Next, 1 μL (2 U) of *E. coli* RNase H was added to each reaction and incubated at 37 °C for 20 min. The resulting cDNA was then stored at −20 °C until used.

### 4.5. qPCR

For qPCR, 100 ng of cDNA was amplified in 20 µL reactions using Fast SYBR Green Master Mix (Applied Biosystems, Waltham, MA, USA, ThermoFisher catalog #4385612). Each reaction was set up in triplicates. ACTB gene served as the endogenous control. qPCR reactions were performed in the QuantStudio™ 7 Flex Real-Time PCR System (Applied Biosystems). Amplification was quantified by SYBR green fluorescence and normalized based on the ROX passive reference dye. Thermocycling program for the amplification reaction was 95 °C: 5 min (95 °C: 15 s, 62 °C: 30 s) × 40 cycles, followed by a melt curve stage (95 °C: 15 s, 60 °C: 1 min, 95 °C: 15 s). Table 2 lists the qPCR specific primer pairs used for the amplification.

### 4.6. Statistical Analysis

Relative expressions of gene transcripts for coculture treatment were measured using ΔCt (the Ct value of the gene of interest normalized to the reference gene), and the data were expressed as the mean ± standard deviation (SD). For comparing gene expression within the same cell line, i.e., GBM and the GBM CSCs, the Fold Change (2^−ΔΔCt^) was measured following the Livak Method. GraphPad Prism 9 software (San Diego, CA, USA) was used for a two-tailed *t*-test in two sample comparisons and one-way ANOVA in group comparisons to estimate statistical significance with Dunnett’s test as the post hoc test. Statistical significance was defined as *p* < 0.05. If the ANOVA showed a significant difference in the variance, then the *t*-test performed was a one-tailed *t*-test. If no difference was detected, then a two-tailed *t*-test was performed.

### 4.7. EV Uptake and Internalization

To visualize the mutual EV uptake, cells were first dyed with fluorescent, lipophilic carbocyanine tracers, green DiO (ThermoFisher Scientific, Waltham, MA, USA, catalog #D3898), and red DiI (ThermoFisher Scientific, catalog #D3886). Right before seeding, cells were treated with 1 uM dye followed by incubation at 37 °C for 5 min [33]. The GBM was dyed red with DiI, whereas the NSCs were dyed green with DiO. EVs secreted by each cell line take the color of the dye of the parent cell. GBM and NSCs were independently cultured for 24 h in a 6-well non-tissue culture treated plate in 4 mL of culture media, and the cells were incubated at 37 °C with 5% CO_2_. The next day, the cells and the conditioned media from each cell line were centrifuged at 0.3 rcf for 5 min at room temperature. The GBM cell pellet was resuspended in NSC-conditioned media, whereas the NSC pellet was resuspended in the GBM-conditioned media with further incubation under the same conditions. The controls did not receive the media switch. After 48 h, the cells were ready to be fixed and visualized using confocal microscopy.

### 4.8. Cell Preparation for Confocal Microscopy

GBM and NSC cells were collected in separate microcentrifuge tubes and centrifuged at 0.3 rcf for 5 min, at room temperature. All subsequent centrifugations were carried out under these conditions unless specified differently. The cell pellet of each cell type was centrifuged again to remove any remaining conditioned media. Pellets were then resuspended in 400 µL of 4% PFA and incubated at room temperature for ~15 min. With another round of centrifugation, PFA supernatant was discarded, and the pellet of fixed cells was washed three times with 500 microliters of ice cold 1X PBS. Then, 5 microliters of the cell suspension in 1X PBS were added as a single drop on a microscope slide. Next, 5 µL of mounting media and DAPI were mixed with each of the cell suspension drops on the microscope slides. A square coverslip was then placed over each drop, and nail polish was used to seal the edges of the coverslip. The microscope slide was allowed to dry in the dark at room temperature.

### 4.9. Confocal Microscopy

Cells were imaged using a Zeiss LSM 710 confocal microscope system with a Zeiss AXIO Observer and the objective plan Apochromat 100x/1.40x Oil DIC M27. The excitation/emission wavelengths are as follows: DiO- 488/542 nm, DiI- 543/675 nm, and DAPI- 358/461 nm. Z-stacks were created for each channel, and a composite image was generated in the image processing program ImageJ.

## 5. Conclusions

The creation of CSC is a dynamic process. Hierarchical versus stochastic/clonal variation models may not be able to describe the process entirely. Tumor microenvironment, epigenetic modifications, and developmental pathways are also likely important factors in driving the process of CSC formation. Factors secreted by NSCs, some of which may be transferred via EVs, may have the capacity to shift the GBM transcriptome towards cancer-specific stemness. On the contrary, GBM-secreted extracellular molecules, diffusible factors and unconventional protein secretions, without association of EVs, may have initiated differentiation in NSCs. Researching each model in detail will advance the understanding of CSC emergence, identify molecular therapeutic targets to eliminate cell resistance, and assist in deciding a distinct therapeutic strategy. Elimination of the CSC population will increase the efficacy of the chemo-radiation treatment, effectively controlling the malignant progression and spread of GBM.

## Figures and Tables

**Table 1:** Relative expressions of gene transcripts detected using qPCR. Delta Ct = [Ct Gene of interest minus Ct ACTB]. One-Way ANOVA was performed to determine the tendency of the variance; based on those results, the corresponding t-test was performed comparing the cell line versus the same cell line with cocultured (CC) conditions.

		Relative Expression (ΔCT)				
		One-Way ANOVA NSC			t-Test CNTRL VS CC	
	GENE	NSC-CC	GBM	GBM-CC	NSC-CC	GBM-CC
ABC transporters	ABCB1	+/−	+/−	+/−	+/−	+/−
	ABCC1	+/−	+/−	− − −	+/−	− − − −
	ABCG2	− − − −	− − − −	+/−	− − −	+ + +
	MGMT	− − −	− − − −	−	− −	+ + + +
Stemness/CSC	TUBB3	−	− − − −	− −	−	+ + +
	CD9	+ + +	− − − −	− − − −	+ +	+ +
	CD133	− − − −	− − − −	+ + +	− − − −	+ +
	CD44	+/−	−	+/−	+/−	+ +
	GFAP	+/−	+ +	+ + +	+/−	+/−
	NANOGP8	+/−	+/−	+/−	+/−	+/−
	SOX2	+/−	+/−	+/−	+/−	+/−
	SOX9	− − −	− − − −	+ + +	− −	+ + + +

No Significance: +/−, Upregulated: +, Downregulated: −, *p* VALUE: <0.05 +; <0.005 ++; <0.001 +++; <0.0001 ++++.

**Table 2:** Primer sequences used in qPCR of the gene transcripts.

	Forward: 5′->3′	Reverse: 5′->3′
ABCB1	CCCATCATTGCAATAGCAGG	GTTCAAACTTCTGCTCCTGA
ABCC1	AACCTGGACCCATTCAGCC	GACTGGATGAGGTCGTCCGT
ABCG2	ATGTCAACTCCTCCTTCTAC	AATGATCTGAGCTATAGAGGC
MGMT	TTCACCATCCCGTTTTCCAG	ATTGCCTCTCATTGCTCCTC
TUBB3	CTCAGGGGCCTTTGGACATC	CAGGCAGTCGCAGTTTTCAC
CD9	GGACGTACTCGAAACCTTCACC	GCGGATAGCACAGCACAAGA
CD133	ACCAGGTAAGAACCCGGATCAA	CAAGAATTCCGCCTCCTAGCACT
CD44	CCAGAAGGAACAGTGGTTTGGC	ACTGTCCTCTGGGCTTGGTGTT
GFAP	ACCTGCAGATTCGAGAAACC	CTCCTTAATGACCTCTCCATCC
NANOGP8	TTTGTGGGCCTGAAGAAAACT	AGGGCTGTCCTGAATAAGCAG
SOX2	TACAGCATGTCCTACTCGCAG	GAGGAAGAGGTAACCACAGGG
SOX9	GCTCTGGAGACTTCTGAACGA	CCGTTCTTCACCGACTTCCT
ACTB	AGAGCTACGAGCTGCCTGAC	AGCACTGTGTTGGCGTACAG

## Data Availability

The data presented in this study are available in the article. The original law data are available on request from the corresponding author.
